# Factors Associated with Non-Adherence to Tuberculosis Preventive Treatment among Adult Contacts of Pulmonary Tuberculosis Cases with Latent Tuberculosis Infection in Catalonia, Spain, in 2019−2021

**DOI:** 10.3390/tropicalmed9030054

**Published:** 2024-02-27

**Authors:** Pedro Plans-Rubió, Sofia Godoy, Diana Toledo, Angela Domínguez, Joan Caylà, Ignasi Parron, Joan Pau Millet, Pere Godoy

**Affiliations:** 1 Public Health Agency of Catalonia, 00805 Barcelona, Spain; 2 CIBER of Epidemiology and Public Health (CIBERESP), Instituto de Salud Carlos III, 28029 Madrid, Spainpere.godoy@gencat.cat (P.G.); 3 Institut Català de la Salut (ICS), 08007 Lleida, Spain; 4 Institut de Recerca Biomèdica (IRB Lleida), University of Lleida, 25006 Lleida, Spain; 5 Department of Medicine, University of Barcelona, 08036 Barcelona, Spain; 6 Barcelona Tuberculosis Research Unit Foundation, 08008 Barcelona, Spain; 7 Epidemiology Service, Public Health Agency of Barcelona, 08023 Barcelona, Spain

**Keywords:** latent tuberculosis infection, tuberculosis, non-adherence to LTBI treatment, factors of treatment non-adherence, community health

## Abstract

The aim of this study was to identify factors associated with non-adherence to tuberculosis (TB) preventive treatment among contacts with latent TB infection for new cases of pulmonary TB cases reported in Catalonia in 2019–2021. All contacts aged 18 years or older with a latent TB infection who received a TB preventive treatment were included in the study. The Chi square test and the odds ratios (OR) were used to assess the association between non-adherence to TB preventive treatment and the study variables; a *p* < 0.05 was considered statistically significant. Multiple logistic regression analysis was used to detect the independent factors associated with TB preventive treatment non-adherence; a *p* < 0.05 was considered statistically significant. The percentage of non-adherence to TB preventive treatment found in this study was 23.7%. A multivariable logistic regression analysis determined that the following factors were significantly associated with TB preventive treatment non-adherence among adult contacts: “exposure at school or workplace” (aOR = 3.34), “exposure to an index case without laboratory confirmation of TB” (aOR = 2.07), “immigrant contact” (aOR = 1.81), “male gender” (aOR = 1.75) and “exposure duration < 6 h per week or sporadic” (aOR = 1.60. By contrast, the factor “short-term TB preventive treatment regimen” (aOR = 0.38) was significantly associated with a lower treatment non-adherence. Adherence to TB preventive treatment should be improved among adult contacts of TB pulmonary cases with latent TB infection by recommending short-term treatment regimens and by developing health education activities, with a greater focus on contacts with factors associated with treatment non-adherence.

## 1. Introduction

Tuberculosis (TB) is transmitted from patients with TB when they expel the bacillus *Mycobacterium tuberculosis* into the air and infect susceptible individuals. Individuals infected with TB bacteria can develop TB disease or a latent TB infection, depending on whether they become sick or not, respectively. TB disease occurs when the immune system cannot avoid the bacteria growth. A latent TB infection occurs when the immune system can avoid TB bacteria growth, but is unable to eliminate TB bacteria from the body. Latent TB infection is therefore characterized by the presence of immune responses to previously acquired *Mycobacterium tuberculosis* infection without clinical evidence of TB disease [1,2]. Most individuals with latent TB infection have no signs or symptoms of TB disease, although they have a 5% risk of developing TB disease in the first 2 years after infection and a 5–20% lifetime risk of developing TB [3]. In 2022, the global reported number of people with newly diagnosed TB was 7.5 million and 1.30 million deaths were caused by TB worldwide [2].

In 2015, all World Health Organization (WHO) and United Nations (UN) Member States committed to ending the TB epidemic through their adoption of WHO’s End TB Strategy and the UN Sustainable Development Goals (SDGs) [4]. The United Nations second high-level meeting on the fight against tuberculosis, held on 22 September 2023, reaffirmed the UN’s commitment to ending the tuberculosis epidemic by 2030 [5]. 

The global targets set in 2023 for the 2023–2027 period included achieving 90% coverage of TB preventive treatment among contacts of pulmonary TB cases and other population groups at high risk of developing TB disease [2]. To achieve this objective, it was considered necessary to treat 20 million household contacts aged ≥5 years during the 2023–2027 period [2]. 

Screening and treating population groups at highest risk of progressing from TB infection to TB disease, such as contacts with latent TB infection of TB cases, is a critical preventive intervention to achieve the global targets of the End TB strategy [6,7,8]. For this reason, the WHO Guidelines on TB [7] and the WHO Guidelines on management of latent TB infection [6,8] proposed to implement the following activities to prevent TB by TB preventive treatment:

(1) Systematic testing and treatment of latent TB infection in adult contacts of pulmonary TB cases.
(2) Systematic testing and treatment of latent TB infection in immigrants from high TB burden countries.
(3) Detection of latent TB infection based on interferon-gamma release assays (IGRA) or the Mantoux tuberculin skin test (TST).
(4) Detection of TB disease in individuals with TB symptoms or radiological abnormalities.
(5) Administration of TB preventive treatments in individuals with latent TB infection.
(6) Clinical monitoring of individuals receiving TB preventive treatment.

Based on the results of the sixth systematic review assessing their efficacy and safety [9], the WHO Guidelines on management of latent TB infection recommended the following TB preventive treatments: 3–4 months rifampicin, 3–4 months isoniazid and rifampicin, 3-month regimen of weekly rifapentine and isoniazid, 6 months of isoniazid, and 9 months of isoniazid [6,7,8]. 

Screening and treating adult contacts with latent TB infection of pulmonary TB cases must be a priority preventive intervention to prevent TB transmission and to achieve the global targets of the End TB strategy, for several reasons. Firstly, the benefits of treatment outweigh the adverse effects [6,7,8,9]. Secondly, the contacts of TB cases have an increased risk of progression from TB infection to TB disease compared to the general population [2,3,6,7,8]. Thirdly, TB preventive treatments can prevent progression from TB infection to TB disease in adult contacts [2,7,8]. Nevertheless, adherence to TB preventive treatment is necessary to achieve treatment effectiveness in preventing progression from TB infection to TB disease and, consequently, to prevent TB transmission in the community [6,7,8,10,11]. A study carried out in Spain found a 11.1% (CI: 5.1–23.3) risk of TB at 5 years among contacts of pulmonary TB cases who did not complete the TB preventive treatment and 1.2% (95% CI: 0.5–3.0) among those who did [10].

In Catalonia, a region in the North-East of Spain with 8 million inhabitants, the incidence of TB was 12.5 cases per 100,000 inhabitants in 2021 [12]. TB incidence increased by 15.7% in 2021 compared with 2020 [12]. Detecting and treating adult contacts of pulmonary TB cases with latent TB infection is a priority public health intervention to prevent TB transmission and reduce TB morbidity and mortality in Catalonia, Spain [2,6,8,11]. The success of this strategy depends on achieving low percentages of TB preventive treatment non-adherence among adult contacts with latent TB infection [4,6,7,11].

The aim of the study was to assess non-adherence to TB preventive treatment and to identify factors associated with treatment non-adherence among adult contacts of pulmonary TB cases with latent TB infection in Catalonia in the 2019–2021 period.

## 2. Materials and Methods

### 2.1. Study Design

A population-based cross-sectional analytical study was performed. Adherence to TB preventive treatment was evaluated among contacts aged 18 years or older of new cases of pulmonary TB registered in Catalonia from 1 January 2019 to 30 June 2021. 

All contacts aged 18 years or older (residing in Catalonia) with a latent TB infection who received a TB preventive treatment were included in the study. The latent TB infection was detected among contacts using the tuberculin test and/or the interferon gamma detection test (IGRA). Contacts with positive IGRA or tuberculin test (≥5 mm) results were considered infected [7,11]. Contacts with a positive test underwent a chest X-ray to detect radiographic anomalies compatible with pulmonary TB. Contacts presenting radiographic anomalies gave a sputum sample to determine the presence of acid-alcohol-resistant bacilli, make a culture, and rule out TB [6,7]. Contacts presenting TB disease were excluded from the study.

### 2.2. Data Collection

A questionnaire was used to collect the following socio-demographic and health information: age, gender, place of birth, place of residence, exposure type, exposure duration, TB preventive treatment, adherence to TB preventive treatment, smoking habit, BCG vaccination, high-risk alcohol consumption, exposure to an index case without laboratory-confirmed TB (smear, culture), or exposure to an index case with pulmonary anomalies compatible with TB detected by computed tomography. 

### 2.3. Data Analysis

Contacts were classified into two groups of TB preventive treatment adherence: (1) fully adherent if they had completed 80% or more of doses and (2) non-adherent if they had completed less than 80% of doses [13,14,15,16,17]. TB preventive treatment regimens were classified into three categories: (1) short-term regimen (rifampicin and isoniazid for three months, rifampicin for four months); (2) long-term regimen (isoniazid for six and nine months); and (3) undefined regimen [6,7,8]. 

The exposure type was classified into four categories, based on the environment where the exposure was produced: cohabiting household, school, workplace, and recreational or ludic. The exposure duration was classified into four categories: daily exposure for 6 or more hours; daily exposure for less than 6 h per day and ≥6 h per week; daily exposure for less than 6 h per week; and sporadic but intense contact (i.e., sharing a car, sharing space in a poorly ventilated place). High-risk alcohol consumption was defined as daily consumption >40 g in men and >24 g in women or a medical record indicating alcohol abuse. Contacts were classified by their smoking habit into four categories: daily smoker, occasional smoker (at least 1 time per week), ex-smoker, and never smoker. 

Percentages and their 95% CI were determined for qualitative variables and means and standard deviations (SD) for quantitative variables. The Chi square test (Fisher’s exact test when necessary) and the Odds Ratios (OR) were used to assess the association between non-adherence to TB preventive treatment and study variables, considering a *p* < 0.05 as statistically significant. The crude ORs were adjusted using multiple logistic regression analysis, considering a *p* < 0.05 as statistically significant.

### 2.4. Bivariate Correlation among Study Variables

Bivariate correlation between TB preventive treatment non-adherence and factors significantly associated with this variable in the univariable analysis and among different significant factors were assessed using the Spearman’s rank correlation coefficient (ρ). A *p* < 0.05 was considered statistically significant. Binary dummy variables were developed for the following variables: TB preventive treatment regimen (short-term regimen vs. long-term/undefined regimen); exposure duration (<6 h per week/sporadic exposure vs. ≥6 h per week); high-risk alcohol consumption (yes vs. no); smoking habit (yes vs. no); exposure type (school/workplace exposure vs. cohabiting household/recreational exposure); contact age (≥30 years vs. <30 years); immigrant contact (yes vs. no); contact gender (male vs. female); exposure to an index case without laboratory TB confirmation (yes vs. no).

### 2.5. Multivariate Logistic Regression Analysis

Multivariable logistic regression analysis was used to detect factors independently associated with non-adherence to latent TB infection treatment. Two multivariable logistic regression models were developed: the full model and the reduced model. The full logistic regression model included all significant variables detected in the univariable analysis and age. The reduced model was developed using the forward variable selection method, using a probability-of-F-to-enter ≤ 0.05 and probability-of-F-to-remove ≥ 0.10. The reduced model was developed to obtain an optimal multivariable model with a lower number of variables than the full model. The statistical analysis was carried out using IBM-SPSS Version 18 (IBM-SPSS, Chicago, IL, USA). 

## 3. Results

### 3.1. Population Studied

**Figure 1:**
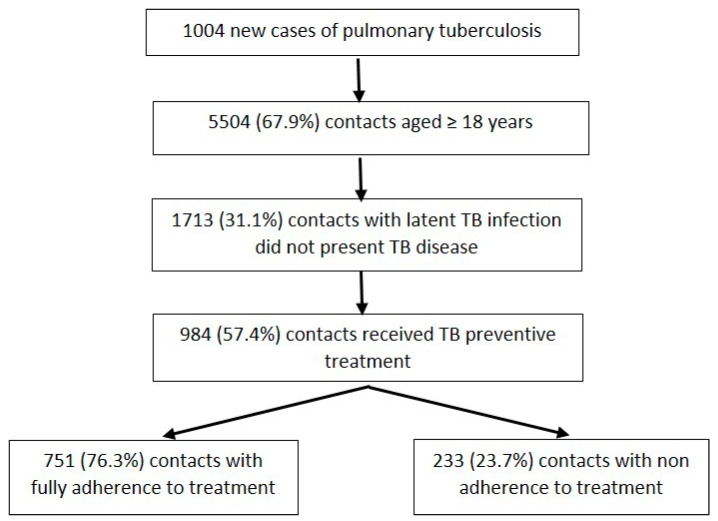
Flux diagram of the study.

A total number of 984 contacts aged 18 years or more who received TB preventive treatment were included in the study (Figure 1).

A total of 1004 new cases of pulmonary TB and their 8107 contacts were registered by the epidemiological services of Catalonia during the study period. Overall, 5504 (67.9%) contacts were aged 18 years or more and 1713 (31.1%) of them tested positive (tuberculin, IGRA) for latent TB infection without TB disease (Figure 1). A total of 984 (57.4%) contacts received a TB preventive treatment. Of these, 751 (76.3%) contacts were fully adherent to TB preventive treatment and 233 (23.7%) were non-adherent to TB preventive treatment (Figure 1). 

### 3.2. Non-Adherence to TB Preventive Treatement

This study found the percentage of TB preventive treatment non-adherence among adult contacts of pulmonary TB cases was 23.7% (95% CI: 21.0–26.4%) (Table 1). A similar mean age was observed among fully adherent contacts (42 years; SD: 14.2) and non-adherent contacts (42.8 years, SD: 13). 

Percentages of non-adherence to TB preventive treatment among adult contacts were significantly higher in the following situations: contacts exposed at school or in the workplace; contacts on short-term treatment regimen; contacts exposed to an index case without laboratory confirmation of TB; contacts with high-risk alcohol consumption; contacts with an exposure duration lower than 6 h per week or sporadic exposure; daily or occasional smokers; immigrant contacts; male contacts (Table 1). 

Percentages of non-adherence to TB preventive treatment were two or more times greater in contacts with exposure at school or in the workplace than in those exposed through cohabitation or a recreational activity; in contacts on a long-term or undefined regimen compared to those on a short-term regimen; and in contacts exposed to an index case without TB laboratory confirmation compared to those exposed to an index case with laboratory confirmation (Table 1). Percentages of non-adherence to TB preventive treatment were less than two times greater in males than in females; in contacts with an exposure duration lower than 6 h per week or sporadic exposure compared to those with an exposure duration lower than 6 h per week; in immigrant contacts compared to autochthonous contacts; in daily or occasional smokers compared to ex-smokers or never smokers; and in contacts with a high-risk alcohol consumption compared to those without a high-risk alcohol consumption (Table 1).

Percentages of non-adherence to TB preventive treatment were higher in contacts aged 30 years or older and contacts vaccinated with the BCG vaccine compared to contacts aged 18–29 years and unvaccinated contacts, respectively, but the differences were not statistically significant (Table 1). 

Percentages of treatment non-adherence in 2019, 2020, and 2021 were slightly different: 23.7% (95% CI: 19.7–27.7) in 2019, 17.6% (95% CI: 11.1–24.0) in 2020 and 24.7% (95% CI: 14.9–34.5) in 2021. Nevertheless, the differences were not statistically significant. 

Contacts with multidrug-resistant TB (MDR-TB) or rifampicin-resistant TB (RR-TB) have not been reported in this study. Adverse effects of latent TB infection treatment were not investigated in this study, but an adverse drug effect was reported for 19 (8.1%) contacts with non-adherence to TB preventive treatment. 

Percentages of non-adherence to TB preventive treatment among contacts without data for the smoking habit (25.3%) and BCG vaccination (21.1%) were not different from the non-adherence percentage found in the study (23.7%). 

### 3.3. Bivariate Correlation among Study Variables

Non-adherence to TB preventive treatment correlated significantly with the following variables (factors): treatment regimen (ρ = −0.17, *p* < 0.001); contact gender (ρ = 0.06, *p* < 0.05); exposure duration (ρ = 0.11, *p* < 0.01); exposure type (ρ = 0.20, *p* < 0.001); high-risk alcohol consumption (ρ = 0.08, *p* < 0.05); smoking habit (ρ = 0.08, *p* < 0.05); immigrant contact (ρ = 0.07, *p* < 0.05); exposure to an index case without laboratory TB confirmation (ρ = 0.11, *p* < 0.01 (Supplementary Materials Table S1). The bivariate correlation between treatment non-adherence and treatment regimen was negative because short-term treatment regimens decreased treatment non-adherence. 

The factor TB preventive treatment regimen correlated significantly with the following variables: exposure type (ρ = −0.07, *p* < 0.05); high-risk alcohol consumption (ρ = −0.15, *p* < 0.001); smoking habit (ρ = −0.35, *p* < 0.001) and immigrant contact (ρ = 0.14, *p* < 0.001) (Supplementary Materials Table S1).

Other significant bivariate correlations were detected between the following variables: exposure type and exposure duration; exposure type and high-risk alcohol consumption; exposure type and smoking habit; exposure type and immigrant contact; exposure duration and high-risk alcohol consumption; exposure duration and immigrant contact; high-risk alcohol consumption and smoking habit; high-risk alcohol consumption and exposure to an index case without laboratory TB confirmation; smoking habit and immigrant contact; smoking habit and exposure to an index case without laboratory TB confirmation; immigrant contact and exposure to index case without laboratory TB confirmation (Supplementary Materials Table S1).

### 3.4. Multivariate Logistic Regression Analysis

The full multivariable logistic regression model, including the statistically significant variables detected in the univariable analysis, determined that only five factors were independently associated with TB preventive treatment non-adherence: treatment regimen, exposure type, index case without laboratory TB confirmation; immigration; and contact gender (Table 2). Exposure in the workplace or at school (aOR = 3.19), exposure to an index case without laboratory confirmation of TB (aOR = 2.16), immigrant contact (aOR = 2.00), and male gender (aOR = 1.56) significantly increased treatment non-adherence, while a short-term TB preventive treatment regimen (aOR = 0.44) significantly reduced treatment non-adherence (Table 2). 

The reduced multivariable logistic regression model determined that six factors were independently associated with TB preventive treatment non-adherence: treatment regimen, exposure type, exposure to an index case without laboratory TB confirmation, immigration, contact gender, and exposure duration (Table 2). The factors “exposure at school or workplace” (aOR = 3.34), “exposure to an index case without laboratory confirmation of TB” (aOR = 2.07), “immigrant contact” (aOR = 1.81), “male gender” (aOR = 1.75), and “low exposure duration” (aOR = 1.60) significantly increased TB preventive treatment non-adherence among adult contacts (Table 2). By contrast, the factor “short-term TB preventive treatment regimen” (aOR = 0.38) significantly reduced treatment non-adherence among adult contacts. 

The forward selection method of the SPSS program introduced the six factors in the model in the following order: exposure type, treatment regimen, immigration, contact gender, index case without laboratory TB confirmation, and exposure duration. The reduced logistic regression model included the factor exposure duration as an independent factor for treatment non-adherence after excluding the variables high-risk alcohol consumption and smoking habit from the model.

## 4. Discussion

Adherence to TB preventive treatment among adult contacts with latent TB infection is necessary for preventing progression from TB infection to TB disease [7,8,11,14]. The study found a percentage of non-adherence to TB preventive treatment of 23.7% among adult contacts of TB pulmonary cases in Catalonia, Spain. The multiple logistic regression analysis revealed that the following six factors increased adherence to TB preventive treatment: “cohabiting household or recreational exposure”, “short-term treatment regimen”, “exposure duration ≥ 6 h per week”, “exposure to an index TB case with laboratory confirmation of TB”, “indigenous contact”, and “female gender”. These factors had a negative effect on treatment non-adherence independently of the effect on the treatment non-adherence of the other factors. By contrast, the factors “exposure at school or workplace”, “long-term or undefined treatment regimen”, “exposure duration < 6 h per week”, “exposure to an index TB case without laboratory confirmation of TB”, “immigrant contact” and “male gender” decreased treatment adherence due to their positive effects on treatment non-adherence. 

The factors “high-risk alcohol consumption” and “smoking habit” were associated with treatment non-adherence only in the univariable analysis. Consequently, their effects on the treatment non-adherence could be explained by their correlations with one of more of the factors included in the multivariable logistic regression models. 

The factor exposure at school or workplace had the highest positive effect on treatment non-adherence, as it increased non-adherence by 234%. The factor index cases without laboratory confirmation of TB increased treatment non-adherence by 107%. The other three factors with a positive effect on treatment non-adherence (immigrant contact, male gender, and low exposure duration) increased treatment non-adherence by 60–81%. By contrast, the factor short-term TB preventive treatment reduced the TB preventive treatment non-adherence by 62%. 

The percentages of TB preventive treatment non-adherence among adult contacts of pulmonary TB cases found in Catalonia, Spain, in this study were similar or higher than those found in studies carried out in Spain [15,16,17,18]. In a study carried out in the province of Lleida in 2016, a non-adherence rate of 29.7% was found in a cohort of 199 contacts (with an average age of 45.1 years) [16]. In a study carried out in the city of Barcelona in 2019, a non-adherence percentage of 29.9% was found in a cohort of 184 infected contacts [17]. In a study carried out in the province of Alicante in 2011, a non-adherence percentage of 19.6% was found in a cohort of 338 contacts (average age of 34.1 years) [17]. In a study carried out in the city of Barcelona by the Vall d’Hebron Hospital in 2018–2020, a non-adherence percentage of 13.4% (95% CI: 9.1–17.7%) was found among 261 (24.1%) contacts [18]. Nevertheless, it is difficult to compare non-adherence rates observed in different studies due to their different methodologies and settings. The study carried out in Alicante included contacts attended by Public Health Services and by the Preventive Medicine Service of Sant Joan Hospital, Alicante, and only tuberculin tests were used to detect latent TB infections [17]. In the study carried out by the Vall d’Hebron Hospital, contacts were treated by the hospital [18]. 

The percentages of TB preventive treatment non-adherence among adult contacts found in this study were similar, greater or lower than the percentages found in other studies carried out worldwide [19,20,21,22,23]. A systematic review and meta-analysis assessing barriers to TB preventive treatment adherence found percentages of non-adherence to TB preventive treatment ranging from 10% to 81% [19]. A prospective cohort study carried out in Norway in 2016 that included 726 individuals notified about TB preventive treatment by the Norwegian Surveillance System for Infectious Diseases found a non-adherence percentage of 9% [20]. A study carried out in Sweden in 2000–2007 found a non-adherence percentage of 24% [21]. A systematic review of studies assessing adherence to TB preventive treatment in the USA and Canada between 1997 and 2007 found non-adherence percentages ranging from 9% to 28% [22]. Nevertheless, it is difficult to compare the non-adherence percentages found in different studies due to differences in study design, study period, setting, population, treatment adherence definition and latent TB infection detection method. 

Previous studies carried out in Spain found similar but not significant results for the factors associated with adherence to TB preventive treatment among contacts of TB cases. In the study carried out in Lleida in 2016, the percentage of adherence to TB preventive treatment was higher in women, autochthonous contacts, cohabitants of the index case and those exposed through cohabitation, although the differences were not statistically significant in the univariable and multivariable logistic regression analyses [16]. The crude ORs for adherence to TB preventive treatment were 1.7 (95% CI: 0.9–3.1) for women, 1.8 (95% CI: 1.0–3.4) for autochthonous contacts, 1.5 (95% CI: 0.8–2.9) for contacts cohabiting with an index case, and 1.4 (95% CI: 0.7–2.9) for household contacts [17]. The adjusted ORs were 1.2 (95% CI: 0.6–2.4) for women, 1.5 (95% CI: 0.7–3.2) for autochthonous contacts, 1.5 (95% CI: 0.9–3.7) for contacts cohabiting with an index case, and 1.4 (95% CI: 0.7–2.9) for household contacts [16]. In the study carried out in the city of Barcelona, TB preventive treatment adherence among immigrants was a little higher compared to that in Spanish-born populations (71.2% vs. 67.8%) but adherence increased to 91.4% for the primary chemoprophylaxis cases [10]. In the study carried out in Alicante in 2011, the adherence to TB preventive treatment was lower in men and immigrant contacts, although the differences were not statistically significant in the univariable and multivariable logistic regression analyses [17]. The crude ORs were 0.6 (95% CI: 0.3–1.2) for men and 0.6 (0.3–1.3) for immigrant contacts. The adjusted ORs were 0.4 (95% CI: 0.2–1.0) for men and 0.8 (0.3–2.1) for immigrant contacts [17]. 

Studies carried out worldwide found factors associated with TB preventive treatment adherence similar to those found in this study (treatment duration, immigrant contact), and factors different to those found in this study, such as absence of perception of risk, alcohol and drug use, and unemployment [11,14,19,20,21,22,23,24,25]. Several studies found that short-term TB preventive treatment regimens were associated with a greater treatment adherence than long-term treatments [14,24,25,26,27]. Nevertheless, other studies did not find significant differences for treatment adherence using different regimens [20]. Currently, short-term TB preventive regimens are recommended based on their similar effectiveness compared with 6–9 months of isoniazid, favorable tolerability, and higher treatment adherence [6,7,8,11,26,27].

Global targets and milestones for reductions in the burden of TB disease, in terms of TB incidence and number of TB deaths, have been proposed by the World Health Organization (WHO) and United Nations (UN) through the adoption of the WHO End TB Strategy (2016–2035) [4,5,28]. In regions such as Catalonia, which have already achieved less than 100 TB cases per million, the TB incidence objective of less than 1 TB case per million people should be achieved by 2050 [29,30]. The European Center for Disease Control and Prevention (ECDC) document on programmatic management of latent TB infection [11], the World Health Organization (WHO) guidelines on TB preventive treatment [7], the WHO guidelines on management of latent TB infection [6,8], the WHO document on the End of TB strategy [28], and the WHO guidelines for programmatic management of latent TB infection [31] consider that contacts of pulmonary TB cases must be a priority population group for developing screening and TB preventive treatment activities. The global targets set in 2023 for the 2023–2027 period included achieving 90% coverage of TB preventive treatment among contacts of pulmonary TB cases and other population groups at high risk of developing TB disease [2]. To achieve this objective, it is necessary to develop a programmatic approach [6,11,31] and to assess the health system, personnel, pharmaceutical and economic resources necessary for its implementation. The programmatic approach includes the following activities: (1) identification of at-risk population groups for testing and treatment of latent TB infection; (2) testing for latent TB infection; (3) detection of TB disease; (4) choosing the TB preventive treatment option that is best suited to each individual; (5) managing the adverse effects of treatment; (6) supporting medication adherence; and (7) monitoring programmatic performance [11,31]. 

The elements of TB prevention and control programs are based on all that is known about the clinical aspects, bacteriology, pathogenesis, epidemiology, prevention, and treatment of the disease. The results obtained in this study showed that more human, pharmaceutical, and economic resources are necessary in Catalonia, Spain, to increase adherence to treatment among contacts of pulmonary TB cases. The identification of factors associated with non-adherence to TB preventive treatment among contacts of pulmonary TB cases is important for guiding prevention activities to increase TB preventive treatment adherence in Catalonia, Spain. Based on the results obtained in this study, the following strategies can be used to increase treatment adherence: (1) using short-term treatment regimens; (2) developing health education activities to improve treatment adherence among adult contacts with latent TB infection. A higher priority for health education activities should be given to the following groups: (1) contacts exposed at school or in the workplace; (2) contacts with an exposure duration of less than 6 h per week; (3) contacts exposed to an index TB case without laboratory confirmation of TB; (4) immigrant contacts; and (5) male contacts. 

In this study, the adherence to TB preventive treatment was lower in contacts exposed to TB cases without laboratory confirmation than in contacts exposed to laboratory confirmed TB cases. This finding suggests that contacts exposed to an index case without laboratory confirmation of TB could have a lower perception of TB risk compared to that for contacts exposed to an index case with laboratory confirmation of TB. For this reason, this group of contacts should have a higher priority for health education activities. All adult contacts included in this study had been exposed to confirmed cases of pulmonary TB. In this study, the TB disease was confirmed in most index cases based on clinical data, laboratory tests, radiography, and epidemiological data [2,7,32], although 9.4% of the contacts included in this study had been exposed to an index case without laboratory TB confirmation. In fact, in 2022, the proportion of people newly diagnosed with pulmonary TB who were bacteriologically confirmed was 91% (IQR: 86−94%) in high-income countries, and it ranged from 71% to 78% in low- and middle-income countries [33]. Other strategies proposed to increase treatment adherence among contacts of TB cases include the following: (1) developing interventions to motivate patients and staff; (2) developing social interventions; (3) developing cultural interventions; and (4) using advanced methods for assessing and monitoring TB preventive treatment adherence, such as direct observed therapy (DOT) [11,14,22,26]. 

This study presents several limitations. Firstly, non-adherence to TB preventive treatment was defined as less than 80% of doses. A definition for non-adherence based on dose-by-dose medication-taking could be more precise [34,35]. Nevertheless, the dose-by-dose data could not be obtained in this study, and full adherence and non-adherence to TB preventive treatments was detected based on the 80% threshold in many studies [13,16,17,36], Secondly, the exposure duration was assessed using a qualitative approach based on four categories, which did not take into account the volume of air shared. However, this approach could make it possible to perform a fast and simple evaluation. Thirdly, this study included all contacts of pulmonary TB cases reported by epidemiological services during the study period. The percentages of non-adherence to TB preventive treatment could be lower or lower than those observed in this study in different population groups if they were under or over reported by epidemiological services, respectively. Nevertheless, the development of a study for assessing whether full adherence (completion) to TB preventive treatment was under or over reported was not possible due to economic and time limitations. 

## 5. Conclusions

In this study, non-adherence to TB preventive treatment among contacts aged 18 years or more with cases of pulmonary TB was 23.7%. The factors associated with non-adherence to TB preventive treatment detected in this study included exposure at school or in the workplace, index cases without laboratory confirmation of TB, immigration, smoking habit, a short exposure duration, and male gender. Adherence to TB preventive treatment should be improved among adult contacts with latent TB infection of TB pulmonary cases by recommending short-term treatment regimens and by developing health education activities, with the higher priority focused on contacts with factors associated with treatment non-adherence. 

## Figures and Tables

**Table 1:** Factors associated with non-adherence to TB preventive treatment among contacts aged 18 years or more of pulmonary tuberculosis cases in Catalonia, Spain, in 2019–2021.

Variable	Values	Non-Adherence to TB Preventive Treatment among Contacts of Pulmonary TB Cases			
		No.	% (95% CI)	OR (95% CI)	n
Age (years)	18–29	47	20.9 (15.3–26.4)	Reference	225
	30–44	75	23.4 (16.6–28.2)	1.15 (0.77–1.75)	320
	45–64	99	25.7 (21.2–30.2)	1.31 (0.88–1.94)	385
	≥64	12	22.2 (10.2–34.2)	1.08 (0.53–2.21)	54
	Total	233	23.7 (21.0–26.4)	–	984
Age (years)	18–29	47	20.9 (15.3–26.4)	Reference	225
	≥30 years	186	24.5 (21.4–27.6)	1.23 (0.86–1.74)	759
Gender	Male	142	26.2 (22.4–30.0) ^+^	1.37 (1.01–1.85)	542
	Female	91	20.6 (16.7–24.5)	Reference	442
TB preventive treatment regimen ^a^	Short-term	51	14.6 (10.7–18.4)	Reference	350
	Long-term	68	26.3 (20.7–31.8) *	2.09 (1.39–3.13)	259
	Undefined	114	30.4 (25.1–32.3) *	2.54 (1.76–3.68)	375
TB preventive treatment regimen ^a^	Short-term	51	14.6 (10.7–18.4)	Reference	350
	Long-term or undefined	182	28.7 (25.1–32.3) *	2.36 (1.67–3.32)	634
Exposure duration	≥6 h per day	119	21.9 (14.8–21.0)	1.26 (0.83–1.92)	544
	<6 h/day and ≥6 h/week	35	18.1 (30.3–41.6)	Reference	193
	<6 h per week	49	37.8 (25.0–26.1) **	2.30 (1.39–3.80)	145
	Sporadic exposure	23	28.0 (16.1–55.2)	1.76 (0.96–3.22)	82
Exposure duration	≥6 h per week	154	20.9 (17.9–23.9) *	Reference	737
	<6 h per week or Sporadic exposure	72	31.7 (25.4–38.0)	1.76 (1.26–2.45)	227
Exposure type	Cohabiting household	110	17.9 (14.8–21-0) *	2.57 (1.88–3.53)	615
	Workplace	106	35.9 (30.3–41.6)	3.36 (1.37–8.25)	295
	Recreational	6	14.3 (2.5–26.1)	Reference	42
	School	10	35.7 (16.1–55.2)	1.01 (0.44–2.27)	28
Exposure type	Cohabiting household or recreational	116	17.7 (14.7–20.6) *	Reference	657
	Workplace or school	116	35.9 (30.5–41.3)	2.61 (1.93–3.53)	323
Immigrant contact	Yes	115	27.3 (22.9–31.7) ^+^	1.42 (1.05–1.90)	421
	No	118	20.9 (17.5–24.4)	Reference	563
Smoking habit	Daily smoker	51	28.5 (21.6–35.4)	2.39 (0.67–8.47)	179
	Occasional smoker	49	24.9 (18.6–31.2)	1.99 (0.56–7.03)	197
	Ex-smoker	3	14.5 (3.0–36.3)	Reference	21
	Never smoker	67	19.8 (15.4–24.2) ^+^	1.48 (0.42–5.18)	338
Smoking habit	Yes	100	26.6 (22.0–31.2) ^+^	1.50 (1.06–2.11)	376
	No	70	19.5 (15.3–23.7)	Reference	359
BCG vaccination	Yes	74	24.9 (19.8–30.0)	1.37 (0.95–1.99)	297
	No	71	19.2 (15.0–23.3)	Reference	370
High-risk alcohol consumption	Yes	19	35.2 (21.5–48.8) ^+^	1.94 (1.08–3.51)	54
	No	147	21.8 (18.6–25.0)	Reference	674
Exposure to an index case without laboratory confirmation of TB	Yes	34	38.2 (27.5–48.9) *	2.16 (1.37–3.42)	89
	No	192	22.2 (19.6–25.3)	Reference	854
Exposure to an index case with pulmonary TB anomalies detected by computed tomography	Yes	49	25.8 (19.3–32.4)	1.06 (0.72–1.55)	190
	No	124	24.7 (20.8–28.6)	Reference	502

OR: odds ratio; CI: confidence interval; TB: tuberculosis; HIV: human immunodeficiency virus; reference: the reference group has an OR = 1. Significance level: * *p* < 0.001, ** *p* < 0.005, ^+^ *p* < 0.05. ^a.^ Short-term regimens included rifampicin and isoniazid for three months and rifampicin for four months. Long-term regimens included isoniazid for six and nine months.

**Table 2:** Multivariable logistic regression analysis assessing the variables explaining the non-adherence to TB preventive treatment among contacts aged ≥18 years of pulmonary TB cases in Catalonia, Spain, in 2019–2021.

Variable	Values Compared	Full Regression Model		Reduced Regression Model	
		aOR (95% CI)	*p*	aOR (95% CI)	*p*
Sex	Men vs. women	1.56 (0.94–2.57)	0.012	1.75 (0.97–2.63)	0.007
Exposure duration	<6 h per week or sporadic vs. ≥6 h per week	1.54 (0.91–2.47)	0.072	1.60 (0.93–2.55)	0.049
Exposure type	Workplace or school vs. cohabiting household or recreational	3.19 (1.57–4.81)	<0.001	3.34 (1.61–5.00)	<0.001
Smoking habit	Yes vs. no	1.41 (0.81–2.25)	0.148	–	–
High-risk alcohol consumption	Yes vs. no	0.93 (0.65–1.92)	0.839	–	–
Immigrant contact	Yes vs. no	2.00 (1.13–3.11)	0.002	1.81 (1.01–2.72)	0.004
TB preventive treatment regimen	Short-term vs. long-term or undefined	0.44 (–0.34–0.71)	0.001	0.38 (–0.49–0.61)	<0.001
Exposure to an index case without laboratory TB confirmation	Yes vs. no	2.15 (1.38–3.98)	0.014	2.07 (1.33–3.76)	<0.001
Constant		0.6 (−2.05–0.13)	<0.001	0.14 (−1.08–0.34)	<0.001

aOR: adjusted odds ratio; CI: confidence interval; TB: tuberculosis.

## Data Availability

The data collected can be made available.

## Supplementary Materials

The following are available online at https://www.mdpi.com/article/10.3390/tropicalmed9030054/s1, Table S1. Bivariate correlation among study variables. The correlation among study variables was assessed using Spearman’s rank correlation coefficient (ρ). Binary dummy variables were developed for the following variables: TB preventive treatment regimen (short-term regimen vs. long-term/undefined regimen); exposure time (<6 h per week/sporadic vs. ≥6 h per week); exposure to high-risk alcohol consumption (yes vs. no); smoking habit (yes vs. no); exposure type (workplace/school vs. cohabiting household/recreational); age (≥30 years vs. <30 years); immigration (yes vs. no); index case without laboratory TB confirmation (yes vs. no); gender (male vs. female).
