# Supplementary material for: Factors Associated with Non-Adherence to Tuberculosis Preventive Treatment among Adult Contacts of Pulmonary Tuberculosis Cases with Latent Tuberculosis Infection in Catalonia, Spain, in 2019−2021
Source: Trop Med Infect Dis. 2024 Feb 27;9(3):54. doi: 10.3390/tropicalmed9030054 (PMC10975791; doi:10.3390/tropicalmed9030054)
Supplement: Supplementary file 1 [file tropicalmed-09-00054-s001.zip › tropicalmed-2855615-supplementary.pdf]

Supplementary material

Table S1. Bivariate correlation among study variables. The correlation among study variables was assessed using the Spearman's rank correlation coefficient (ϱ). Binary dummy variables were developed for the following variables: TB preventive treatment regimen (short-term regimen vs. long-term/undefined regimen); exposure time (<6 hours per week/sporadic vs. ≥6 hours per week); exposure to high-risk alcohol consumption (yes vs. no); smoking habit (yes vs. no); exposure type (workplace/school vs. cohabiting household/recreational); age (≥30 years vs. <30 years); immigration (yes vs. no); index case without laboratory TB confirmation (yes vs. no); gender (male vs. female).

Variable		Non-adherence to TB preventive treatment	Gender	Exposure duration	Exposure type	High-risk alcohol consu-mption	Smoking habit	Immi-gration	Index case without laboratory TB confir-mation	Age
Gender	ϱ	0.066								
	p value	0.039								
	n	984								
Exposure duration	ϱ	0.108	−0.007							
	p value	0.001	0.825							
	n	964	964							
Exposure type	ϱ	0.202	0.052	0.263						
	p value	<0.001	0.101	<0.001						
	n	980	980	964						
High-risk alcohol consumption	ϱ	0.084	−0.162	0.081	0.097					
	p value	0.024	<0.001	0.030	0.009					
	nn	728	728	723	728					
Smoking habit	ϱ	0.084	−0.012	0.047	0.178	0.202				
	p value	0.023	0.749	0.208	0.000	<0.001				
	n	735	735	729	735	728				
Immigration	ϱ	0.074	0.161	−0.076	−0.082	−0.021	−0.359			
	p value	0.020	<0.001	0.018	0.010	0.564	<0.001			
	n	984	984	964	980	728	735			
Index case without laboratory TB confirmation	ϱ	0.109	0.080	−0.044	0.025	0.093	−0.125	0.170		
	p value	0.001	0.014	0.176	0.441	0.013	<0.001	<0.001		
	n	953	953	933	949	702	709	953		
Age	ϱ	0.036	0.043	0.047	0.020	0.054	−0.023	−0.004	0.023	
	p value	0.263	0.173	0.141	0.536	0.146	0.530	0.910	0.487	
	n	984	984	964	980	728	735	984	953	
TB preventive treatment regimen	ϱ	−0.168	0.061	−0.050	−0.075	−0.151	−0.353	0.136	0.034	−0.048
	p value	<0.001	0.059	0.126	0.021	<0.001	<0.001	<0.001	0.303	0.136
	n	984	952	932	948	714	952	952	922	952
